# Current Status and Significance of Additional Vaccination with COVID-19 Vaccine in Japan—Considerations from Antibody Levels from Hybrid Immunity and Public Perceptions

**DOI:** 10.3390/vaccines12121413

**Published:** 2024-12-15

**Authors:** Hiroshi Kusunoki

**Affiliations:** Department of Internal Medicine, Osaka Dental University, 8-1 Kuzuhahanazonocho, Hirakata 573-1121, Osaka, Japan; kusunoki1019@yahoo.co.jp

**Keywords:** COVID-19, vaccine, hybrid immunity

## Abstract

This report examines the evolving role of coronavirus disease 2019 (COVID-19) vaccination in Japan, especially in light of the reduced public concern following the reclassification of COVID-19 as a Category 5 infectious disease in May 2023. With over half the population estimated to have hybrid immunity from prior infections and vaccinations, this report evaluated the necessity and frequency of additional booster doses. Despite strong recommendations from Japanese medical societies to continue vaccination, public skepticism remains owing to financial burdens, adverse reactions, and the perceived limited benefits of frequent boosters. Studies on antibody responses have revealed that individuals with hybrid immunity maintain robust protection with significantly elevated antibody titers that persist over extended periods. Case studies have indicated durable immunity among individuals who have both been vaccinated and experienced breakthrough infections, raising questions about the need for uniform booster policies. This report also discusses the newly approved replicon-type (self-amplifying) vaccines currently available only in Japan, which have generated public and professional debates regarding their efficacy and safety. A more personalized approach to vaccination that takes into account the antibody titers, prior infection history, and individual choices is recommended. Finally, this report underscores the importance of aligning vaccination policies with scientific evidence and public sentiment to optimize COVID-19 countermeasures in Japan.

## 1. Introduction

Nearly five years have passed since coronavirus disease 2019 (COVID-19) was first reported in Wuhan, China, in 2019. The so-called “COVID-19 crisis”, during which COVID-19 dominated societal concerns, persisted for some time. However, since the reclassification of COVID-19 into Category 5 under Japan’s Infectious Disease Control Law in May 2023, public interest in the virus appears to have waned, and society seems to have regained a sense of normalcy. Mask usage has declined, and the public now regards the “COVID-19 crisis” as a matter of the past. The author is pleased to see that society has finally returned to a normal state.

On the other hand, COVID-19 vaccination efforts will continue in the fall of 2024, with vaccines available to high-risk groups, including adults over 65 years of age and individuals aged 60–64 years who have certain underlying conditions (such as heart, kidney, or respiratory dysfunctions that significantly impair daily life and immune dysfunction caused by HIV). These groups are eligible for routine vaccination [1].

In response, the Japanese Society of Infectious Diseases, in association with the Japanese Respiratory Society and the Japanese Society for Vaccinology, has issued a “Position Paper on Routine COVID-19 Vaccination in 2024”, strongly recommending routine vaccination with the COVID-19 vaccine in fall/winter 2024, as COVID-19 is considered more severe and has a worse prognosis than influenza [2]. Additionally, the Japan Pediatric Society recommends vaccination for all children aged 6 months to 17 years, including initial and booster doses, at appropriate intervals [3]. The Pharmaceuticals and Medical Devices Agency (PMDA) also emphasized the efficacy and safety of the COVID-19 vaccine [4]. Prominent infectious disease experts continue to feature in newspaper advertisements sponsored by the vaccine manufacturers, advocating for additional vaccinations [5,6].

However, as vaccination is now voluntary and individuals bear most of the costs (although some local government subsidies are available), there are concerns. Past vaccinations led to unexpected severe reactions, such as fever, and some individuals experienced lasting side effects. Moreover, with the perception that COVID-19 has become less severe and that infections typically result in mild symptoms, the number of people expected to receive the COVID-19 vaccine in Japan in the fall/winter of 2024 may be significantly less than anticipated.

This review aims to assess the necessity and frequency of additional COVID-19 booster vaccinations in Japan, with a focus on the antibody levels sustained through hybrid immunity, which combines vaccination and natural infection. Additionally, it explores the public’s evolving perceptions of vaccination, shaped by concerns over financial burdens, adverse reactions, and the perceived limited benefits of frequent boosters. By analyzing recent trends and scientific evidence, this review seeks to propose a more personalized and evidence-based approach to vaccination policies in Japan. While this review article references numerous online sources, the author wishes to emphasize that these citations are intended solely to confirm objective facts and are not meant to defame or criticize any specific individual or group.

## 2. Hybrid Immunity and Its Implications for Public Health Policy

A study conducted using residual blood samples from blood donors to assess severe acute respiratory syndrome coronavirus 2 (SARS-CoV-2) antibody prevalence revealed that as of 31 March 2024, the prevalence of anti-N (nucleocapsid) antibodies, indicative of previous SARS-CoV-2 infection, was 64.5% (64.2% among males and 64.7% among females) [7]. This prevalence is 80% higher among individuals aged 16–19 and generally decreases with age, although the majority of those aged 60–69 already harbor these antibodies. Given that COVID-19 was also prevalent during the summer of 2024, the existing antibody prevalence is likely to be even higher. Considering the anticipated recurrent waves of COVID-19 in the coming summers and winters, the prevalence is expected to approach nearly 100% over the next several years.

Antibodies against SARS-CoV-2, particularly those targeting the receptor-binding domain (RBD) of the spike protein, play a critical role in public health. RBD antibodies inhibit the virus’s ability to enter host cells, thereby preventing infection. The concentration of these antibodies serves as an indicator of an individual’s immune status and can be used to assess the persistence of immunity over time.

Moreover, measuring RBD antibodies is essential in evaluating the effectiveness of vaccines. The production of RBD antibodies following vaccination indicates the induction of immunity against SARS-CoV-2. Investigating the relationship between RBD antibody levels and actual infection prevention helps clarify the duration of vaccine efficacy and the need for booster doses. Additionally, understanding the spread of RBD antibodies within a population provides valuable information to guide strategies for preventing the transmission of SARS-CoV-2.

The combination of vaccination and natural SARS-CoV-2 infection, referred to as “hybrid immunity”, is known to elicit a particularly robust immune response, with significantly increasing antibody titers [8,9,10,11,12]. To monitor IgG antibody responses to the receptor-binding domain (RBD) of the S1 subunit of the SARS-CoV-2 spike protein, we used the Abbott Architect SARS-CoV-2 IgG II Quant chemiluminescent microparticle immunoassay (Abbott Laboratories, Chicago, IL, USA) in a limited cohort of outpatients. In cases of natural infection following vaccination (commonly termed ‘breakthrough infection’), hybrid immunity led to a notable increase in RBD antibody levels [13,14,15]. During the one-year follow-up period, we observed that antibody titers remained elevated, although they gradually decreased over time [16]. Additionally, in cases of natural infection occurring in the summer of 2023, after five vaccine doses, antibody titers increased to over 80,000 AU/mL, exceeding the assay’s measurable limit. Furthermore, in some individuals, the titers remained in the tens of thousands of AU/mL for over 6 months.

Since most individuals previously infected with COVID-19 in Japan experienced breakthrough infections after receiving two or more vaccine doses, and with more than 80% of the Japanese population having received at least two vaccine doses, it is estimated that a significant (likely a majority) population has acquired hybrid immunity and sustains high antibody titers. Consequently, the necessity for frequent additional vaccinations, as recommended in Japan through 2023, warrants further consideration.

In the United States, a comprehensive study of beneficiaries within the U.S. Military Health System was conducted to identify predictors of vaccine immunogenicity. This study demonstrated that compared with vaccine- or infection-only immunization, hybrid immunity was associated with sustained antibody titers at 6 months post-infection. The researchers reported that antibody titers remained elevated for at least 6 months following hybrid immunization, potentially informing optimal vaccination timing strategies [17]. Similarly, studies involving New York City residents indicated that the rate of antibody titer decline over time was lower among residents with hybrid immunity [18]. More recently, a study in China revealed a gradual decline in anti-SARS-CoV-2 IgG antibodies following breakthrough infections, indicating that the vaccination efficacy persisted for over a year [19].

## 3. Case Studies of Antibody Responses Following Vaccination and Infection

**Figure 1:**
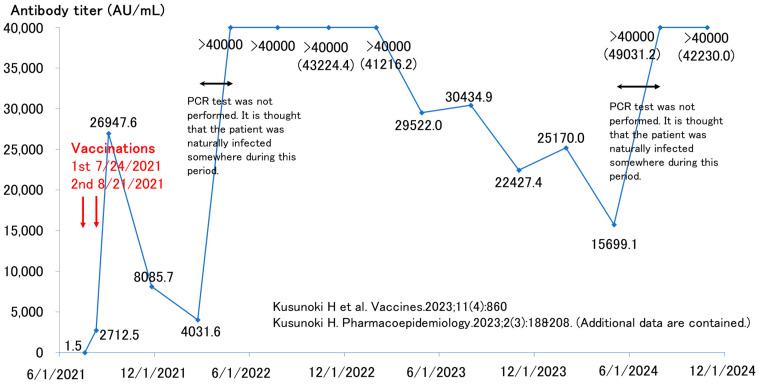
Antibody titer trends in a male in his 40s [13,15] (Additional data are contained).

Figure 1 illustrates the antibody response in a man in his 40s. Before his initial vaccine dose in July 2021, he tested negative for antibodies and had no prior suspected COVID-19 infection. After receiving the second dose, his antibody levels increased to nearly 27,000 AU/mL but decreased to approximately 4000 AU/mL over the following 6 months. In April 2022, his antibody titers unexpectedly increased to above 40,000 AU/mL, well beyond the measurable limits. The antibody levels gradually declined but spiked again to approximately 40,000 AU/mL between May and July 2024.

At the time of the first increase in antibody titer in April 2022, he was considering receiving his third vaccination in the near future. This suggests that he was likely infected with COVID-19 between February and March 2022, although no PCR test was performed and thus it was not documented as a COVID-19 case [13,15]. The second rise in antibody titers in July 2024 suggests that he was likely reinfected in the two to three months immediately preceding that time. Throughout this period, he experienced no symptoms, such as fever, and remained untested, indicating a probable asymptomatic infection.

**Figure 2:**
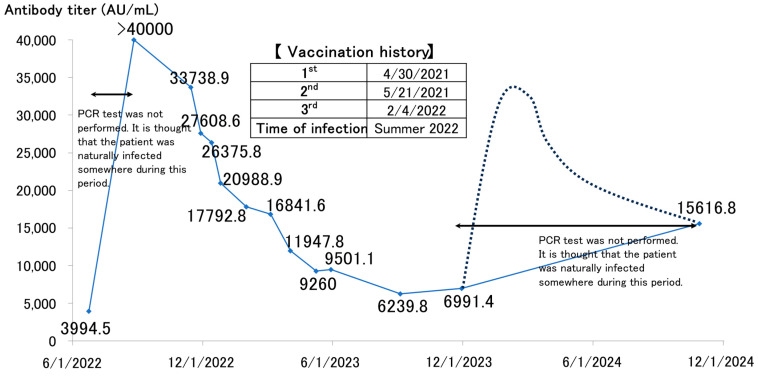
Antibody titer trends in a male in his 60s [16] (Additional data are contained).

Figure 2 depicts a man in his 60s who was likely to have contracted COVID-19 spontaneously in the summer of 2022, resulting in an antibody titer increase to >40,000 AU/mL (the upper limit of our facility’s measurements at that time). Although titers decreased over time, a plateau was seen, remaining within the 6000–7000 AU/mL range. In October 2024, antibody titers were reassessed after approximately a year. An increase of more than 15,000 AU/mL was seen.

Subsequent antibody measurements revealed a non-linear decline, following an exponential decay pattern, f(x) = e^−x^. The antibody titer in the 6000–7000 AU/mL range, which decayed to a plateau, is comparable to the levels observed immediately after the second or third vaccination. This level of antibody retention indicated that the titers resulting from hybrid immunity remained impressively high.

The re-increase in antibody titers seen in 2024 suggests at least one natural infection in 2024. Given his history of reaching >40,000 AU/mL post-infection, there is a possibility that his antibody levels initially increased significantly before stabilizing at 15,000 AU/mL (indicated by the dotted line in the Figure 2). Such cases, with asymptomatic or mild infections and no confirmatory tests, are likely to contribute to community transmission. Owing to these instances, COVID-19 transmission surges twice yearly, in summer and winter, making it virtually impossible to curtail the virus’s spread during these periods.

## 4. Epidemiological Trends in Antibody Titers and Vaccine Efficacy

Our study, though based on a small number of cases, highlights the need for a large-scale study in Japan to investigate the trends in COVID-19 antibody titers over time, particularly in post-vaccination breakthrough cases, which currently constitute the majority of COVID-19 infections in Japan.

An epidemiological study in Bizen City, Okayama Prefecture, conducted by Okayama University, revealed that antibody titers sustained for longer in individuals with prior SARS-CoV-2 infection than in uninfected individuals following booster doses [20,21]. This study also measured antibodies against the RBD of the spike protein and analyzed the relationship between antibody titer levels and infection risk. The non-linear correlation observed indicated that infection risk decreased with increasing antibody titers, although the slope of risk reduction flattened around the 10,000 AU/mL mark [22,23].

**Figure 3:**
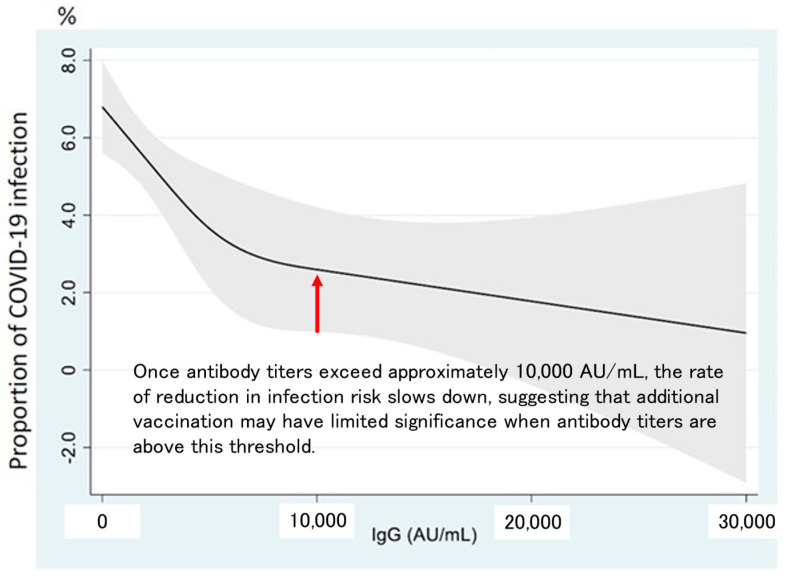
Association between IgG antibody titers against the RBD of the spike protein (AU/mL) and the risk of SARS-CoV-2 infection (%) [22]. The natural cubic spline that had three knots (2500, 5000, and 10,000 AU/mL)is shown as a black line and the 95% confidence intervals (CIs) are shown as a gray range.

This finding is particularly significant, as epidemiological data in Japan suggest that once antibody titers surpass 10,000 AU/mL, additional vaccination has minimal benefit in further reducing the risk of infection (Figure 3). In other words, for individuals who have achieved antibody titers of 10s of 1000s of AU/mL through hybrid immunity, additional vaccination may have limited value. Further, considering adverse reactions such as fever or thrombotic risk, this could pose a higher risk than benefit. Given that a large proportion of the Japanese population has already attained high antibody levels through hybrid immunity, a uniform recommendation for additional vaccination remains highly questionable. In the future, scheduling vaccination may need to be adjusted more flexibly, considering factors such as age, underlying health conditions, antibody titer levels, and timing of prior infections.

## 5. Global Trends in COVID-19 Vaccination Uptake: A Focus on Japan’s Exceptional Rates

In fact, many countries outside Japan have ceased offering additional vaccinations after 2022. For instance, VIVALDI, a prospective cohort study conducted in long-term care facilities in England, showed that while successive booster doses provided short-term protection against COVID-19-related mortality, relative to the third booster dose, no added long-term benefit from the fourth or fifth doses was noted [24]. This underscores the fact that additional vaccinations beyond the third dose may not always be beneficial.

However, in the United States, the Advisory Committee on Immunization Practices (ACIP) recommended on 12 September 2023, that every person aged 6 months and older receive at least one dose of the 2023/24 COVID-19 vaccine. Despite this recommendation, as of 31 March 2024, only 22.6% of eligible adults and 14% of children (aged 6 months to 17 years) had received the vaccine [25].

**Figure 4:**
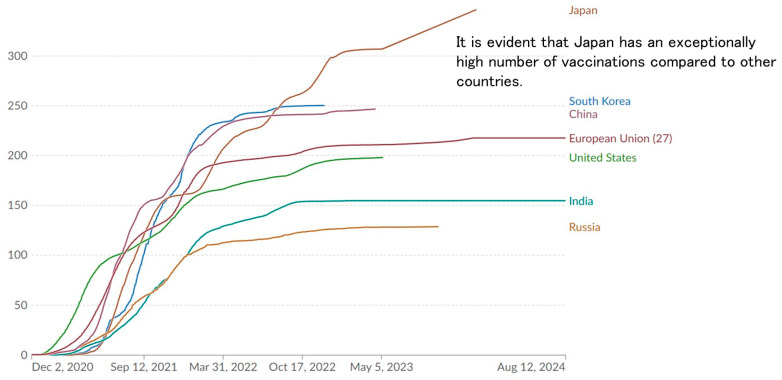
Total COVID-19 vaccine doses per 100 individuals. https://ourworldindata.org/ (accessed on 9 December 2024).

Figure 4 illustrates the cumulative number of COVID-19 vaccinations per 100 population by major country, including additional booster doses. It is evident that the number of vaccinations in Japan is significantly higher than in India, Russia, Europe, and the United States, as well as in China and South Korea, which are also located in East Asia and have relatively high vaccination rates.

## 6. Analysis of Vaccine Recommendations by Japanese Medical Societies

In their position paper on routine COVID-19 vaccination in 2024, the Japanese Society of Infectious Diseases and other academic organizations strongly recommended routine COVID-19 vaccination for older adults in the fall and winter of 2024. They reference a study claiming that the vaccine has prevented 14.4 million COVID-19-related deaths worldwide [26]. However, this study provided a one-year estimate beginning in December 2020, when mRNA vaccines were first introduced, and may thus not reflect the current conditions. Many studies cited as evidence for vaccine effectiveness tend to describe its impact from the time it was initially launched. Additionally, organizations argue that COVID-19 remains more severe and fatal than influenza, claiming that COVID-19 deaths are approximately three times more than those from influenza. An online article with the headline “COVID-19 deaths are about 15 times more common than flu deaths” also fueled public perceptions [27].

While influenza peaks in winter, COVID-19 circulates year-round, with notable surges in summer and winter. SARS-CoV-2 has largely replaced coronaviruses that caused the common cold before the pandemic. With frequent SARS-CoV-2 antigen testing for the common cold and fever, many patients with typical cold symptoms are now diagnosed with COVID-19. Even when COVID-19 is not the primary cause of death, a positive test result may still classify death as COVID-19-related, potentially contributing to an apparent increase in COVID-19 deaths. Therefore, a comparison of the risks between the two is not possible.

Another cited reason for strongly recommending vaccination is the risk of post-acute sequelae (Long COVID). Severe symptoms persisting over an extended period, commonly referred to as Long COVID, indeed appear to exist in a subset of cases. Among people aged 70 and older, 15.7% reported symptoms lasting over 3 months that disrupted daily life [28]. However, these post-COVID symptoms were self-reported, and objective measures were not used in the assessments. In a cross-sectional study utilizing questionnaires and electronic health records, over half of the patients reported persistent COVID-19 symptoms of various severities one year post-infection [29]. Participant enrollment for this study occurred in 2020 at an early pandemic stage when COVID-19 symptoms were likely to be different from those associated with the Omicron variant. The authors also noted that selection and recall biases could have influenced participants’ willingness to engage in the study, possibly resulting in an overestimation of Long COVID prevalence, as those experiencing prolonged symptoms were more inclined to participate. Given that 60–70% of the entire population has now been infected with COVID-19, it seems unlikely that the true prevalence of Long COVID is as high as these studies suggest. Thus, current estimates of Long COVID rates may be inflated.

## 7. Evaluating the Justification for Additional SARS-CoV-2 Vaccinations

The vaccine effectiveness real-time surveillance for SARS-CoV-2 (VERSUS) study was primarily conducted by Nagasaki University, recognized as a leader in infectious disease research in Japan. The university has sent a significantly larger number of board members to the Japanese Society of Infectious Diseases than other universities. However, opinions suggest that the VERSUS study may include or exclude cases arbitrarily to suit its purpose [30]. According to the findings, while the effectiveness of additional vaccination in preventing severe disease was significant, its effectiveness in preventing disease onset was not as pronounced [31,32].

If the efficacy of preventing the development of Long COVID is not substantial, even in the presence of a high incidence of Long COVID, the recommendation for additional vaccinations may be less justified in terms of their effectiveness against this condition.

Some may argue that individuals who have been infected in the past may exhibit a weak immune response to the current epidemic strain, necessitating vaccination against the latest variants. Indeed, if more than a year has passed since a person’s previous infection, protection against the current pandemic strain may be inadequate. In these cases, additional vaccinations may be beneficial. However, individuals who were naturally infected in the summer of 2024 are likely to have developed sufficient immunity against the updated strain of the virus, diminishing the significance of further vaccination.

## 8. Reflecting on Japan’s Response: Successes, Challenges, and Public Trust

Many people in Japan believe that “COVID-19 was brought under control due to the proper advice of experts, the hard work of medical personnel, and the leadership of politicians. Above all, the citizens were very cooperative in implementing infection control measures, including masks and vaccines. The vaccine was also immensely effective, saving many lives” [33].

Considering that Japan has spent over 300 trillion yen on COVID-19 countermeasures, and that the Japanese population has had to bear significant economic and mental burdens due to self-restraint and other measures, leading to immense sacrifices across various areas, the author finds himself inclined to agree with this sentiment.

However, with regards to the cost-effectiveness of additional doses, as mentioned by us, the necessity of frequent booster vaccinations is highly questionable. Therefore, it may be necessary to reconsider whether aggressive recommendations are truly appropriate, particularly for young individuals and healthy adults. In some instances, excessive infection control measures and booster recommendations proposed by experts can cause confusion. The author has previously noted the widespread distrust of infectious disease specialists among the general public [16]. Negative opinions about infectious disease experts, infection control measures, and additional vaccinations tend to be particularly pronounced in online spaces such as Yahoo! Comments. While this stream of comments does not necessarily reflect the views of the public as a whole, a significant number of people are skeptical of the infection control measures and vaccines promoted by infectious disease specialists.

Conversely, while politicians often face criticism, their leadership in organizing the Tokyo Olympics without spectators, allowing individuals to decide whether to wear masks, and classifying COVID-19 as a class 5 disease, despite the reluctance of infectious disease experts, should perhaps be more highly regarded. Had they strictly adhered to the recommendations of Dr. Shigeru Omi and his colleagues, the infectious disease experts central to Japan’s COVID-19 response, the economic and social disruptions would likely have been even greater. Moreover, the fact that many citizens cooperated in wearing masks and receiving vaccinations may have been due to strong social pressure and a sense of collective responsibility among their peers rather than a high level of public awareness.

Japan has experienced a population decline since the COVID-19 pandemic [34], with a particular increase in excess deaths becoming a topic of discussion. Some reports have suggested that the COVID-19 vaccine may be associated with an increase in deaths [15,19,35,36]. In an interview in September 2024, Dr. Shigeru Omi stated, “Unfortunately, Japan does not have a system in place to obtain detailed data on vaccine-induced damage and deaths. The situation is such that we hardly know whether the cause of the deaths was the vaccine or something else, meaning we cannot draw any conclusions at this time. I believe Japan should quickly establish a monitoring system for close scrutiny” [37,38]. Although Dr. Omi has made efforts to gain public trust, such as appearing on social media [39], his words and actions have been described as highly cowardly [40]. While establishing such a monitoring system would be challenging owing to budgetary constraints and the risk of arbitrary data manipulation, it is possible to investigate and publish data on the incidence, severity, and mortality rates relative to the number of vaccine doses administered, even through retrospective studies. This should be pursued as soon as possible, as anti-vaccine groups might argue that the reluctance to conduct such a study stems from fear of uncovering negative findings about COVID-19 vaccines.

Employees of vaccine manufacturing companies actively publish studies emphasizing the efficacy and safety of vaccines while advocating for higher vaccination rates. In Japan, a study conducted by Pfizer employees estimated COVID-19-related symptoms, hospitalizations, deaths, and both vaccine- and non-vaccine-related medical costs and productivity losses at vaccination rates of 50%, 90%, and 10%, respectively. The study found that even with increased vaccine-related costs, overall medical expenses and productivity losses exceeded those costs. The authors concluded that non-vaccine-related medical costs and productivity losses could be significantly reduced, which could lead to lowering overall costs and underscoring the importance of maintaining high vaccination coverage among the elderly and at-risk populations [41]. However, further validation of these estimates is essential because the results may vary considerably depending on the assumptions and premises of the models employed.

Additionally, a review article by a Moderna employee in the U.S. highlighted the low rate of additional COVID-19 vaccinations in the U.S. and recommended that all individuals aged 6 months and older should receive further vaccinations. The article emphasized the importance of integrating vaccination into routine medical care and establishing consistent vaccination guidelines by healthcare providers to encourage COVID-19 vaccination [25].

The reasons cited for the low uptake of additional vaccinations include a lack of long-term safety data and efficacy information, a decreased sense of urgency for COVID-19 vaccination compared to the earlier stages of the pandemic, and insufficient communication about the need for additional doses and updates to vaccination schedules from healthcare providers and the general public. However, there has been minimal discussion regarding the lack of scientific evidence supporting the necessity and frequency of additional vaccinations.

Fundamentally, because vaccine manufacturers have a direct economic interest in increasing vaccination rates, the fairness and objectivity of studies published by their employees are questionable, and some may argue that these studies are inappropriate in terms of conflicts of interest.

## 9. The Role of Replicon Vaccines: New Developments and Controversies

The COVID-19 vaccination program for 2024 that commenced in October had a significant attention focused on a replicon-type (self-amplifying) mRNA vaccine developed by Arcturus Therapeutics in the United States and Meiji Seika Pharma in Japan. This vaccine has been shown to induce higher neutralizing antibody titers than conventional mRNA vaccines [42], with an advantage of a longer duration of neutralizing antibodies [43,44].

However, widespread skepticism emerged, particularly among those already doubtful of existing COVID-19 vaccines, regarding why this vaccine was approved solely in Japan and not in the U.S. where it was developed, or Vietnam, where clinical trials were conducted. Additionally, employees of Meiji Seika Pharma published a book revealing the inner workings of the pharmaceutical company [45]. The theory that recipients of the replicon vaccine can shed the virus and infect others has gained traction, leading to frequent bans on replicon vaccine recipients entering doctors’ offices, beauty salons, and other venues. Meiji Seika Pharma is currently preparing to sue a member of the Diet who has repeatedly made negative remarks about the replicon vaccine, accusing him of “inciting public anxiety by misrepresenting facts and raising issues not grounded in scientific knowledge” [46].

Disseminating unsubstantiated information emotionally and sensationally is not appropriate. Nevertheless, given that the replicon vaccine represents a novel vaccine type that has not yet been approved in other countries, many individuals express concerns about this vaccine. Additionally, negative opinions from critics of replicon vaccines emerged in various online articles [47].

Regarding “unnecessarily inflaming public fears”, the words and actions of infectious disease experts in the early stages of the pandemic that contributed to public anxiety are worth noting. In 2020, a prominent infectious disease expert announced that over 420,000 people could die without intervention, heightening public anxiety. As the results of such large-scale estimates are sensitive to even marginal changes in assumptions, a thorough verification of these assumptions and a reassessment based on multiple scenarios should have been conducted, with the findings made accessible to the general public. Furthermore, questioning the credibility and realistic applicability of such figures is reasonable because they may be perceived as disconnected from the experiences of the general public and clinicians. Experts also estimated that without the introduction of the new COVID-19 vaccine, the number of cases and deaths would have been 13.5 times and 36.4 times higher, respectively, than those reported between February and November 2021 [48]. These data also seem far removed from the perceptions of many citizens and clinicians, and some presentations have raised questions about the validity of these findings [49,50,51].

Similar episodes have been reported in other studies. Nobel Prize laureate Dr. Shinya Yamanaka noted not only the efforts of Japanese health authorities and the public’s self-restraint in reducing outings and decreasing close contact through practices such as handshaking and kissing compared to Western cultures, but also pointed to public health programs such as childhood BCG vaccinations against tuberculosis. He suggested that the Japanese people may possess genetic resistance to SARS-CoV-2 or have already acquired resistance through infections with similar viruses. Collectively, these factors were referred to as “Factor X”, sparking considerable controversy [52]. Some specialists have argued that the idea of Japanese resistance to COVID-19 is merely an illusion [53] and that Factor X does not exist [54,55].

Concerns may have arisen regarding the risk that “Factor X” could provide the public with a false sense of security, leading to the neglect of other essential infection prevention measures. These specialists may have asserted that “Factor X” was an illusion lacking scientific basis and evidence. However, stating that “Factor X” is an illusion might have left the public with the impression that “biological differences” attributed to it are impossible, which may reflect a lack of prudence on the part of scientists regarding unnecessary public anxiety.

Despite aggressive recommendations from academic societies and vaccine manufacturers, the demand for the COVID-19 vaccine is expected to decline in the fall/winter of 2024. Many individuals, including the elderly, are likely to refrain from receiving the COVID-19 vaccine, partly because of the reduction in public subsidies for the vaccine and the proliferation of various negative perceptions. With a large supply of vaccines already available (over 30 million doses), a significant surplus will likely need to be disposed of. Furthermore, it is unlikely that individuals who have already declined the vaccination will reconsider their decisions in the future, even beyond the fall/winter of 2025. Although this situation poses challenges for vaccine manufacturers, who have made substantial capital investments anticipating a high demand for COVID-19 vaccines, it would be prudent to order a realistic number of vaccines post-2025 based on the projected vaccination population for 2024.

Opinions regarding COVID-19 vaccines and infection control measures are sharply divided between proponents and opponents, often resulting in irreconcilable arguments. This dichotomy frequently leads to disputes that emphasize differences rather than deepening the discussion. While both sides may deny each other’s claims, it is essential to respect the coexistence of differing opinions and promote an approach that values individual choices in policy decisions. Both sides present reasonable arguments, but there are also emotional, aggressive, and excessive aspects as well as points that raise doubts. Both sides also have a tendency to disseminate information selectively, emphasizing what is most convenient for their respective positions. In discussions surrounding the COVID-19 vaccine, even mild criticism tends to label individuals as “anti-vaccine” or conspiracy theorists, while information that is slightly negative about the vaccine is often dismissed as misinformation.

## 10. Limitations and Future Directions in COVID-19 Vaccination Research in Japan

This review highlights the importance of adopting a more individualized and evidence-based approach to COVID-19 vaccination policy in Japan. By focusing on antibody levels sustained through hybrid immunity—achieved via a combination of vaccination and natural infection—and analyzing the current social context in Japan alongside recent trends in medical research, we emphasize the need for tailored vaccination strategies.

One limitation of this review is its focus solely on antibodies against the receptor-binding domain (RBD) of the spike protein, without addressing neutralizing antibodies. Neutralizing antibodies bind to viral surface proteins and inhibit infection and transmission by preventing viral interaction with human cell receptors [56]. Despite their critical role in immunity against COVID-19, the measurement of neutralizing antibodies requires considerable time and specialized cell culture facilities, making large-scale testing impractical [57]. Since IgG antibodies targeting the RBD strongly correlate with neutralizing antibodies [58,59], the evaluation of SARS-CoV-2 immunity based on RBD-specific IgG antibodies is currently deemed reasonable.

Another limitation is the lack of discussion on the role of natural immunity against SARS-CoV-2 following vaccination. In the context of hybrid immunity, the influence of natural immunity is significant. Studies have demonstrated that natural immune responses, including CD4+ T cells, CD8+ T cells, and memory B cells, persist for an extended period after COVID-19 infection [60,61]. It has also been reported that the protective effects of innate immunity against SARS-CoV-2 infection diminish over time, though the prevention of severe cases remains robust [62].

In the future, it would be desirable for Japan to conduct large-scale studies on cases of acquired hybrid immunity, with a particular focus not only on humoral immunity but also on natural immunity, including cellular immunity. Given Japan’s unprecedented number of COVID-19 vaccinations, the country is well-positioned to undertake highly significant research in this area. Continuing this type of rigorous research will contribute to restoring confidence in the field of infectious disease and, by extension, in the broader medical profession and healthcare community.

## 11. Concluding Thoughts on COVID-19 Vaccination Policies and Future Directions

The author believes that the COVID-19 vaccine represents an excellent technological advancement, as significant increases in antibody titers can be achieved with the first two doses, particularly when accompanied by a spontaneous infection. In healthy individuals, the risk of severe illness is minimal, and the author does not completely dismiss the efficacy of the COVID-19 vaccine. The fact that there will be a positive impact during the initial vaccination period in 2021, and in some cases, the benefits of additional vaccinations may still outweigh the risks, is acknowledged.

Furthermore, the author respects the autonomy of patients and healthcare providers who choose to receive additional vaccinations and does not advocate against their decisions. This choice reflects a form of personal freedom that must be honored. However, those who opt for additional vaccinations should not criticize or pressure individuals who are hesitant or unwilling. As for-profit entities, vaccine manufacturers have the right to promote their products actively.

Nonetheless, for the author to be labeled “anti-vaccine” by those around them, including family members, merely for questioning the need for additional vaccinations, has been a painful experience. In Japanese culture, there is a tendency to act based on societal expectations and peer pressure rather than rational judgment. Looking ahead, it is crucial to develop policies that respect individual values and choices regarding vaccination.

## 12. Conclusions

The COVID-19 pandemic has profoundly affected Japan’s healthcare system, societal norms, and public policies. With the development of hybrid immunity across a significant portion of the population, there is a compelling need to re-evaluate the blanket approach to booster vaccinations. Evidence from domestic and international studies suggests that hybrid immunity confers strong and long-lasting protection, potentially reducing the need for additional vaccinations. Therefore, vaccination strategies should be adapted considering individual immune status, including antibody levels and infection history, to promote a more targeted and cost-effective approach.

Public skepticism toward continued vaccination highlights the broader need for transparent communication from healthcare authorities. Vaccine manufacturers and policymakers must recognize the importance of public trust and address concerns regarding vaccine safety, cost, and effectiveness. Furthermore, Japan’s experience underscores the importance of balancing expert guidance with flexibility in public health policies to foster resilience against future health crises.

Moving forward, a policy framework that respects individual choices and is informed by ongoing scientific data is crucial. By embracing a nuanced approach that considers the diverse immunity profiles of its population, Japan can optimize its COVID-19 strategy, ensuring that public health measures are both scientifically grounded and publicly supported.
